# Синдром Ван Вика-Громбаха как результат поздней диагностики аутоиммунного тиреоидита у пациентки с синдромом делеции 22-й хромосомы. Описание клинического случая и краткий обзор литературы

**DOI:** 10.14341/probl13555

**Published:** 2025-12-02

**Authors:** А. А. Момотова, Т. Е. Иванникова, А. В. Витебская, Ю. В. Тихонович

**Affiliations:** Первый Московский государственный университет им. И.М. Сеченова (Сеченовский университет)Россия; I.M. Sechenov First Moscow State Medical University (Sechenov University)Russian Federation; Первый Московский государственный университет им. И.М. Сеченова (Сеченовский университет); Российский национальный исследовательский медицинский университет им. Н.И. Пирогова; Медико-генетический научный центр им. академика Н.П. БочковаРоссия; I.M. Sechenov First Moscow State Medical University (Sechenov University); Russian National Research Medical University named after N.I. Pirogov; Medical Genetic Research Center named after Academician N.P. BochkovRussian Federation

**Keywords:** синдром делеции 22 хромосомы, синдром Ди Джорджи, синдром Ди Георга, синдром Ван Вика-Громбаха, гипотиреоз, раннее половое развитие, chromosome 22 deletion syndrome, Di Giorgi syndrome, Di Georg syndrome, Van Wyk-Grombach syndrome, hypothyroidism, early sexual development

## Abstract

Синдром делеции 22-й хромосомы (22q11.2 DS, del22q11.2) (при выраженных иммунологических нарушениях — синдром Ди Георга (СДГ), или синдром Ди Джорджи (СДД)) является одним из наиболее часто встречающихся микроделеционных синдромов.

В основе заболевания лежит нарушение формирования органов, происходящих из третьей жаберной дуги. Различают полную форму синдрома del22q11.2 с тяжелым первичным иммунодефицитным состоянием (ПИД), врожденными пороками сердца (ВПС), гипопаратиреозом, аномалиями лицевого скелета и высокой летальностью в течение первого года жизни и частичные формы без ПИД и нарушений кальций-фосфорного обмена.

Высокая вариабельность клинических проявлений объясняет тот факт, что в литературе имеется множество различных наименований заболевания: синдром Ди Джорджи (СДД), синдром Ди Георга (СДГ), CATCH 22, велокардиофасциальный синдром, синдром Кайлера, синдром Шпринтцена, синдром лицевых и конотрункальных аномалий и т.д. Термин «синдром Ди Джорджи» применим к случаям делеции 22q11.2 хромосомы, протекающими с иммунными нарушениями.

Несмотря на доступность генетического тестирования, многие случаи синдрома делеции 22q11.2 остаются недиагностированными из-за его мультисистемного характера и различной выраженности клинических проявлений, что сопряжено с высоким риском развития жизнеугрожающих осложнений.

Мы приводим данные пациентки 9 лет с частичной формой синдрома делеции 22q11.2, когда поводом для обращения к эндокринологу стало раннее появление вторичных половых признаков на фоне декомпенсированного первичного гипотиреоза (синдром Ван Вика-Громбаха) при отсутствии нарушений фосфорно-кальциевого обмена и ПИД. Представленный клинический случай демонстрирует не только вариабельность клинической симптоматики данного заболевания, но и необходимость слаженного взаимодействия специалистов различных специальностей для диагностики полиморфной хромосомной патологии.

## Abstract (en)

22nd chromosome deletion syndrome (22q11.2 DS, del22q11.2) (with severe immunological disorders – Di Georg syndrome (SDH) or Di Giorgi syndrome (SDD)) It is one of the most common microdeletion syndromes.

The disease is based on a violation of the formation of organs originating from the third gill arch.

There is a full form of del22q11.2 syndrome with severe primary immunodeficiency (PID), congenital heart defects (CHD), hypoparathyroidism, facial skeletal abnormalities and high mortality during the first year of life, and partial forms without PID and calcium-phosphorus metabolism disorders.

The high variability of clinical manifestations explains the fact that there are many different names of the disease in the literature: Di Giorgi syndrome (SDD), Di Georg syndrome (SDH), CATCH 22, velocardiofacial syndrome, Kyler syndrome, Sprintzen syndrome, facial and conotruncal abnormalities, etc.

The term «Di Giorgi syndrome» is applicable to cases of deletion of 22q11.2 chromosome occurring with immune disorders. Despite the availability of genetic testing, many cases of 22q11.2 deletion syndrome remain undiagnosed due to its multsystem nature and varying severity of clinical manifestations, which is associated with a high risk of life-threatening complications.

We present data from a 9-year-old patient with a partial form of deletion syndrome 22q11.2, when the reason for contacting an endocrinologist was the early appearance of secondary sexual characteristics against the background of decompensated primary hypothyroidism (Van Wyk-Grombach syndrome) in the absence of violations of phosphorus-calcium metabolism and PID.

This clinical case demonstrates not only the variability of the clinical symptoms of the disease, but also the need for coordinated interaction of specialists from various specialties to diagnose polymorphic chromosomal pathology.

## АКТУАЛЬНОСТЬ

Синдром делеции 22-й хромосомы (синдром del22q11.2) — одна из самых распространенных хромосомных аномалий, возникающая вследствие делеции длинного плеча одной копии 22-й хромосомы. Делеция хромосомы 22q11.2 является самой частой причиной СДД, реже СДД возникает в результате перестроек других хромосом или мутаций в гене TBX1 [1].

Частота встречаемости синдрома del22q11.2 в различных популяциях составляет от 1:3000 до 1:6000 новорожденных [2][3].

В подавляющем большинстве случаев заболевание возникает спорадически, в 10% случаев наследуется от одного из родителей (аутосомно-доминантный тип наследования) [4].

Первые упоминания о пациентах с фенотипом СДД относятся к 1828 г. Полное описание синдрома принадлежит доктору Анджело Ди Джорджи, который в 1965 г. описал пациентов с аплазией тимуса и паращитовидных желёз [5].

Позже к списку ключевых признаков СДД были добавлены ВПС, поддерживая теорию о том, что развитие заболевания связано с нарушением формирования органов, происходящих из 3 жаберной дуги [6].

В начале 1990-х гг. исследования гибридизации флуоресценции in situ (FISH) с использованием зондов в удаляемой области выявили субмикроскопические делеции 22q11.2 как наиболее частую причину СДД [7].

В результате этого открытия появился термин «Синдром делеции 22 хромосомы», объединяющий целый ряд состояний с единым генетическим механизмом и схожими фенотипическими особенностями [8].

Клиническая картина заболевания вариабельна иногда даже в пределах одной семьи.

В современной иммунологии практикуется разделение СДД на полный и частичный. Критерии полного СДД включают в себя тяжелый Т-клеточный иммунодефицит с выраженным снижением или полным отсутствием CD3+Т-клеток в результате гипоплазии/аплазии тимуса (75%), гипопаратиреоз (50%), ВПС (75%). Термин «Частичный синдром Ди Джорджа» подразумевает сочетание типичных клинических признаков без выраженного иммунодефицита [9][16].

Летальность у детей с делецией 22 хромосомы на первом году жизни составляет около 4–5%, фатальные исходы чаще ассоциированы с ВПС, гипокальциемией и развитием трахеомаляции [2].

В качестве наиболее частых причин преждевременной смерти у взрослых пациентов фигурируют острая сердечно-сосудистая недостаточность, инсульт, злокачественные новообразования, пневмония, суицид [10].

Несмотря на широкую доступность генетического тестирования, многие случаи СДД остаются недиагностированными из-за его мультисистемного характера и различной выраженности клинических проявлений. В некоторых случаях диагноз взрослым пациентам устанавливается только после рождения в семье больного ребенка. В настоящее время в литературе описаны более 180 клинических форм синдрома делеции 22-й хромосомы и более 190 симптомов заболевания, которые могут затрагивать практически все органы и системы [11][12].

Наиболее частым эндокринным проявлением СДД является гипопаратиреоз, реже встречаются патология щитовидной железы, задержка роста, ожирение и т.д. [3].

Мы приводим данные пациентки 9 лет с неполной формой делеции 22 хромосомы, когда поводом для обращения к эндокринологу стало раннее появление вторичных половых признаков на фоне декомпенсированного первичного гипотиреоза при отсутствии нарушений фосфорно-кальциевого обмена и ПИД.

## МАТЕРИАЛЫ И МЕТОДЫ

Клинико-лабораторное обследование пациентки проводилось в отделении детской эндокринологии (ДЭ) Сеченовского центра материнства и детства (СЦМиД), г. Москва. Генетическое исследование было проведено в частной лаборатории по месту жительства ребенка.

## ОПИСАНИЕ СЛУЧАЯ

Пациентка 9 лет была направлена в детское эндокринологическое (ДЭ) отделение СЦМиД с жалобами на прогрессирующий набор веса, снижение темпов роста, запоры, сухость кожных покровов, утомляемость, снижение успеваемости в школе. Из анамнеза известно, что ребенок от первой беременности, протекавшей с угрозой прерывания, срочных, самостоятельных родов. Вес при рождении — 2750 г (SDS ИМТ –0,73), рост — 47 см (SDS +0,45). Во время беременности у матери был диагностирован аутоиммунный тиреоидит (АИТ), назначена терапия левотироксином натрия. Рост мамы — 164 см, отца — 182 см. Прогнозируемый средне-родительский рост ребенка — 166,5 см.

После рождения у пациентки были диагностированы множественные пороки развития: ВПС (дефект межпредсердной перегородки, дефект межжелудочковой перегородки — прооперирована в 5 месяцев жизни, в настоящее время получает курсами верошпирон); атрезия ануса; ректо-вагинальный свищ (прооперирована в 1,5 года). С раннего возраста отмечались низкие темпы роста, отставание в психомоторном развитии (ходит с 1,5 года, речь с 3 лет), дизартрия, назолалия, снижение слуха, частые респираторные инфекции, требующие антибактериальной терапии.

С 7,0–7,5 года появились жалобы на прогрессирующий набор веса, утомляемость, снижение успеваемости, запоры, увеличение молочных желез. Получала симптоматическую терапию, гормональный профиль не исследовался.

В 8 лет 5 мес родители обратились к эндокринологу в связи с прогрессирующим увеличением молочных желез, отечностью лица и век. Выявлены гиперпролактинемия, повышение уровня ТТГ>100 мЕд/л, антител к ТПО — до 278 МЕ/мл. Также отмечалось повышение эстрадиола до 135 пмоль/л при допубертатных значениях ЛГ и ФСГ. Данные обследования пациентки представлены в таблице 1.

**Table:** Таблица 1. Данные обследования пациентки

	8 лет 5 месяцев Первичное обращение	8 лет 5 месяцев Госпитализация 1	8 лет 8 месяцев	9 лет Госпитализация 2	9 лет 3 месяца
Антропометрические данные	-	Рост — 118 см (SDS роста: -1,68) Вес — 26,9 кг (SDS ИМТ + 1,32)	-	Рост — 120,5 см (SDS роста: -1,72) Вес — 24,2 кг (SDS ИМТ + 0,14)	-
Левотироксин, мкг/сутки	-	25	50	62,5	62,5
свТ4, пмоль/л (12,5–21,5)	5,41	6,5	14,5	23,2	19,23
ТТГ, мкМЕ/мл (0,6–4,84)	> 100	747,1	39,6	2,92	0,79
АТ-ТПО, МЕ/мл (0–34)	278	289	-	113	-
Кортизол, нмоль/л (119–618)	-	263	-	191	-
Пролактин, мкМЕ/мл (42–912)	2249	2311	232	132	-
Биоактивный пролактин, мкМЕ/мл (109–557)	1739	-	-	-	-
ИФР-1, нг/мл (99–376)	85,1	433	-	-	-
ЛГ, мМЕд/мл	<0,09	0,1	0,1	0,2	-
ФСГ, мМЕд/мл	3,31	2,2	0,3	0,3	-
Эстрадиол, пмоль/л	135	< 18,35	<18,35	<18,35	-
ПТГ, пмоль/л (1,48–7,63)	-	-	-	0,5	1,4
25-ОНД, нг/мл (30–100)	23	-	-	-	65,0
Са иониз., ммоль/л (1,15–1,35)	-	-	-	1,28	1,19
Са общ. ммоль/л (2,2–2,7)	2,34	2,42	2,38	2,34	2,12
Р (1,03–1,87) ммоль/л	1,65	1,6	1,4	1,65	1,8
АЛТ (N 10:35) ед/л	149	45	12	8	-
АСТ (N 0:35) ед/л	123	39	24	23	-

По данным МРТ головного мозга с контрастным усилением, выявлено эндо-супраселлярное объемное образование достаточно однородной структуры, размером 8*15*23 мм. На основании клинико-лабораторных данных установлен диагноз: синдром Ван Вика-Громбаха на фоне декомпенсированного гипотиреоза вследствие АИТ, рекомендован прием левотироксина натрия в стартовой дозе 25 мкг/сутки. Для дальнейшего обследования девочка направлена в отделение ДЭ СЦМиД.

При поступлении в отделение через 10 дней после начала приема левотироксина обращали на себя внимание сухость кожных покровов, избыток массы тела, укорочение шеи, увеличение нижней трети лица, пастозность лица и век, полуптоз, удлиненное лицо, широкий и выступающий корень носа, широкая переносица, низко посаженные ушные раковины (рис. 1), брадикардия и брадипное (ЧДД 20 в мин., ЧСС 64 в мин., АД 90/60 мм рт.ст.) Щитовидная железа визуально и пальпаторно не увеличена, клинически — гипотиреоз. Половое развитие Танер 2 (В2P2), менструации отсутствовали.

**Figure:**
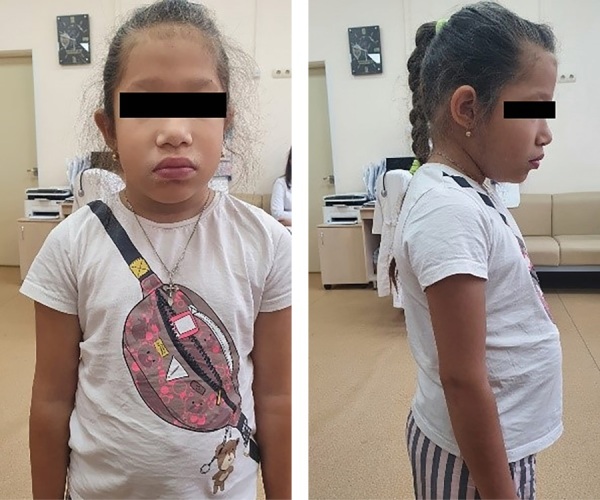
Рисунок 1. Пациентка при первой госпитализации.

По результатам биохимического и гормонального исследования сохранялись выраженный гипотиреоз и гиперпролактинемия, эстрадиол снизился до допубертатных значений (табл. 1). По данным пробы с аналогом гонадотропин-рилизинг гормона (ГН-РГ) максимальный выброс ЛГ составил 0,3 мМЕ/мл, что исключало гонадотропинзависимый характер преждевременного полового развития.

По данным УЗИ, щитовидная железа не увеличена (объем — 5,1 см³), выявлены характерные для АИТ изменения структуры по типу «тяжистости».

При проведении УЗИ органов малого таза обращало на себя внимание увеличение яичников (правый 56х32х36 мм, левый 52х29х34 мм) с множественными фолликулами (до 10–12 в срезе) до 18 мм в диаметре.

Костный возраст по атласу TW20 cоставил 7,5 года при паспортном возрасте 8,5 года.

МР-снимки были проконсультированы в ФГАУ НМИЦ имени Н.Н. Бурденко, подтверждена аденома гипофиза, которая была расценена как следствие длительного повышения секреции ТТГ и пролактина на фоне первичного гипотиреоза.

На основании полученных данных пациентке был установлен диагноз:

«Аутоиммунный тиреоиодит, первичный декомпенсированный гипотиреоз. Синдром Ван Вик-Громбаха. Объемное образование гипофиза (вторичная гиперплазия на фоне гипотиреоза?). Гипохромная анемия. Тугоухость 1 ст. Ангиопатия сетчатки. Дисфункция гепатобилиарного тракта. Состояние после хирургического лечения ВПС (пластика дефекта межпредсердной перегородки, дефекта межжелудочковой перегородки) в 2014 г. Частичная реканализация дефекта межпредсердной перегородки, диаметр шунта до 8 мм. Умеренный стеноз легочной артерии».

Попытка увеличения дозы левотироксина до 50 мкг/сутки сопровождалась развитием тахикардии до 120–130 в минуту, в связи с чем к моменту выписки из отделения доза препарата снижена до 37,5 мкг/сутки. В дальнейшем девочка продолжала наблюдаться амбулаторно с коррекцией заместительной терапии на основании результатов гормонального профиля (табл. 1).

Принимая во внимание особенности фенотипа, несоответствие фактического роста ребенка среднеродительскому, множественные врожденные пороки развития (ВПР), наличие патологии щитовидной железы, заподозрена неполная форма синдрома делеции 22q.11.21.

По результатам полноэкзомного секвенирования получены данные в пользу наличия гетерозиготной делеции региона 22q.11.2, захватывающей в том числе ген TBX1. В дальнейшем делеция 22 хромосомы была подтверждена методом MLPA с использованием набора реактивов P250-B2 DiGeorge probemix: обнаружена делеция NC_000022.10:g.(?_19241636)_(21349221_?)del (GRCh37/hg19), включающая в себя регион LCR22-A-D, в гетерозиготном состоянии. Данный патогенный вариант был раннее описан в гетерозиготном состоянии у пациентов с синдромом Ди Джорджи и велокардиофациальным синдромом.

С родителями ребенка были обсуждены риски развития гипопаратиреоза, рекомендован контроль показателей кальций-фосфорного обмена, осмотр отоларинголога в связи с наличием назолалии, консультация иммунолога для исключения ПИД.

При обследовании через 6 месяцев на фоне регулярного приема левотироксина в дозе 62,5 мкг в сутки отмечена хорошая динамика роста, нормализация веса и ранее измененных лабораторных показателей (табл. 1, рис. 2, рис. 3).

**Figure:**
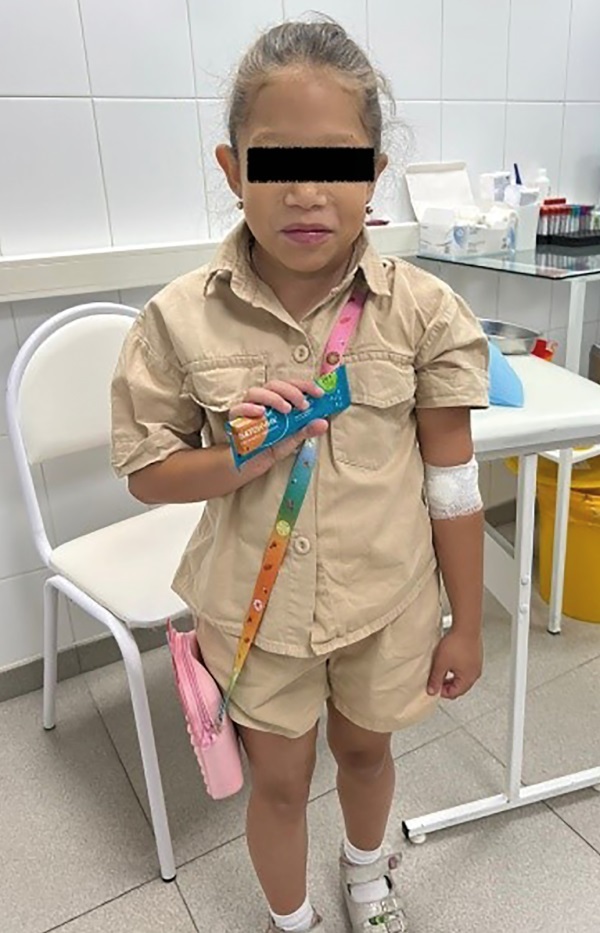
Рисунок 2. Пациентка через 6 месяцев на фоне регулярного приема левотироксина.

**Figure:**
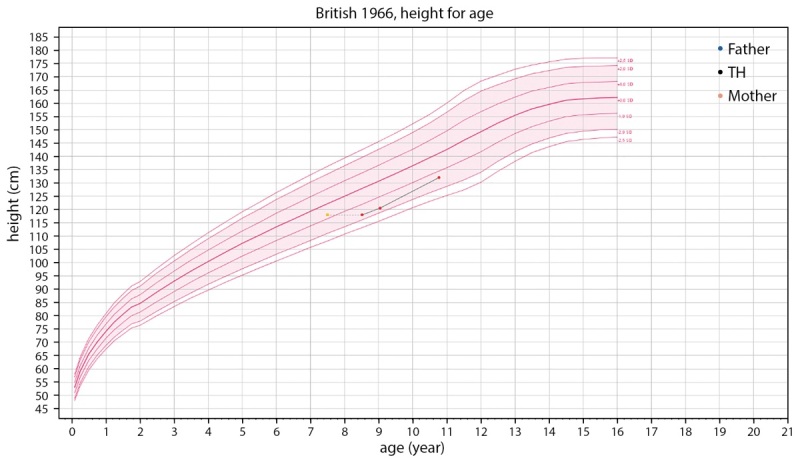
Рисунок 3. Кривая роста пациентки.

Впервые выявлено снижение уровня паратгормона до 0,5 пмоль/л (1,48–7,63), при этом показатели кальций-фосфорного обмена находились в пределах референсных значений, в связи с чем пациентке был рекомендован прием неактивной формы витамина Д3 в профилактической дозировке, наблюдение в динамике.

По результатам МРТ головного мозга и гипофиза, проведенного в 9 лет на фоне терапии левотироксином, микроаденома (в левой половине гипофиза отмечается очаг 2,4 мм с пониженным МР-сигналом в Т1ВИ, после контрастного усиления — не накапливающий контрастный препарат).

По поводу дизартрии, назолалии, пациентка консультирована отоларингологом, выявлен еще один ВПР — скрытая расщелина нёба, по поводу которой в 9,3 года проведено оперативное лечение.

## ОБСУЖДЕНИЕ

Полиморфизм клинических проявлений при синдроме делеции 22 хромосомы связан с «поломкой генома» в области q11.2 длинного плеча хромосомы 22. Типы и размеры делеций демонстрируют высокую степень вариабельности из-за нескольких повторяющихся последовательностей с низким числом копий (LCR22A, LCR22B, LCR22C, LCR22D, LCR22E и LCR22F) [13]. У большинства пациентов (около 90%) обнаруживается гетерозиготная делеция размером примерно 3 Мб, включающая около 46 генов, что приблизительно соответствует 3 млн пар нуклеиновых оснований [14]. В 10–12% случаев встречаются более короткие делеции, которые составляют 1,5–2 млн парных оснований [15][16].

Согласно литературным данным, тяжесть клинических проявлений не коррелирует с размером делеции, кроме того, фенотипические признаки могут варьировать даже в пределах одной семьи [17][18].

Наиболее изученным геном в области делеции 22q11.2 является TBX1, кодирующий фактор транскрипции T-box.

Регулируя экспрессию факторов роста и факторов транскрипции, участвующих в развитии сердца, тимуса, паращитовидной железы и неба, TBX-1 является ведущим геном, ответственным за фенотипические признаки заболевания (рис. 4) [19].

**Figure:**
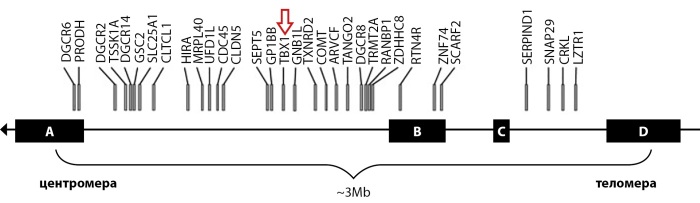
Рисунок 4. Гены в типичной области делеции 22 хромосомы (адаптировано из статьи «Hacıhamdioğlu B, Hacıhamdioğlu D, Delil K. 22q11 deletion syndrome: current perspective. Appl Clin Genet. 2015 May 18; 8: 123-132») [19].

Менее 1% пациентов с микроделецией 22q11.2 имеют классический фенотип с тяжелым ПИД, ВПС и гипопаратиреозом. Без трансплантации гемопоэтических клеток такие пациенты погибают в течение первого года жизни [20].

Термин «Частичный синдром Ди Джорджа» используется у пациентов, если они имеют сочетание типичных признаков, но без ПИД [21].

Дополнительные проявления синдрома включают пре- и постнатальную задержку роста, пороки развития мочеполовой системы, пищеварительного тракта, нёбные аномалии (явная расщелина нёба, скрытая подслизистая расщелина нёба, раздвоенный язычок, небно-глоточная дисфункция), снижение слуха, лицевой дизморфизм, сколиоз, полидактилию, микроцефалию, мышечную гипотонию, когнитивный дефицит, особенности поведения и т.д. (табл. 2) [22].

**Table:** Таблица 2. Основные клинические проявления синдрома Ди Джорджи [3][9][22][23][24][26][27]

Фенотип	Ретрогнатия, удлиненное лицо, прямой профиль лица, зубные аномалии, гипертелоризм, косоглазие, уплощение скул, короткие глазные щели, опущенные веки, широкий и выступающий корень носа, широкая / раздвоенная переносица, низкая переносица, выпуклый кончик носа, микрогнатия, маленький рот, неправильно сформированные, маленькие, низко посаженные уши, удлиненные пальцы
Иммунологические проявления	Гипоплазия или аплазия тимуса. Нарушение выработки Т-клеток, у 6% — дефицит IgA, у 23% — гуморальные дефекты, рецидивирующие инфекции, аутоиммунные заболевания
Сердечно-сосудистая система	Тетрада Фалло, ДМЖП, транспозиции магистральных сосудов, прерывистая дуга аорты и двойной выходной отдел правого желудочка
Эндокринная система	Гипопаратиреоз, задержка роста, ожирение, СД2, АИТ, рак щитовидной железы, азооспермия. Пороки развития щитовидной железы (отсутствие перешейка щитовидной железы, ретрокаротидное и ретропищеводное расширение, отсутствие / гипоплазия левой доли щитовидной железы)
Небные аномалии	Верхнеглоточная недостаточность, подслизистая расщелина неба, волчья пасть, последовательность Пьера Робина, раздвоенный язычок и велофарингеальная дисфункция
Патология желудочно-кишечного тракта	Гастроэзофагеальная рефлюксная болезнь (ГЭРБ), нарушение моторики пищевода, носоглоточный рефлюкс, атрезия прямой кишки, болезнь Гиршпрунга, атрезия пищевода или трахео-пищеводный свищ
Нарушения со стороны мочеполовой системы	Двусторонняя или односторонняя агенезия почек, диспластические или кистозные почки, гидронефроз
Психические расстройства	Тревожность, дефицит внимания, расстройства аутистического спектра (РАС), биполярное расстройство, шизофрения
Аномалии нижних дыхательных путей	Трахео- и бронхомаляция, стеноз трахеи, короткая трахея с редуцированными трахеальными кольцами, аберрантный трахеобронхит, трахеопищеводный свищ
Аутоиммунные заболевания	Ювенильный ревматоидный артрит (ЮРА), витилиго, идиопатическая пурпурная тромбоцитопения, гемолитическая анемия, аутоиммунная нейтропения, апластическая анемия, целиакия
Офтальмологические особенности	Извитые сосуды сетчатки, птоз, аномалия Аксенфельда-Ригера, склерокорнея, колобома, катаракта, анофтальмия и косоглазие
Стоматология	Задержка прорезывания зубов, агенезия постоянных зубных рядов, гипоплазия эмали, нарушение кальцификации эмали
Нарушения со стороны ЦНС	Уменьшение лобных, височных, теменных и затылочных долей головного мозга, атрофия мозжечка и гиппокампа, полимикрогирия и изменение толщины коры, повышенный риск болезни Паркинсона
Аномалии скелета	Затылочно-шейная аномалия, сколиоз, аномалии ребер и позвонков, косолапость, полидактилия

К наиболее частым эндокринным проявлениям синдрома относятся гипопаратиреоз, патология щитовидной железы и задержка роста. Гораздо реже встречаются ожирение, сахарный диабет (СД), нарушения полового развития, азооспермия [23][24].

Так, Chen X. и соавт. описали случай первичного гипогонадизма у взрослого мужчины с синдромом делеции 22 хромосомы в результате двусторонней атрофии яичек [25].

Гипопаратиреоз, обусловленный гипоплазией или аплазией паращитовидных желез, является ведущим эндокринным проявлением СДД и встречается в 60–65% случаев заболевания. По данным литературы, гипопаратиреоз чаще встречается в неонатальном периоде и в первые годы жизни ребенка, хотя описаны случаи манифестации заболевания у подростков и взрослых пациентов [3][26].

Первичный гипотиреоз при синдроме делеции 22q11.2 развивается вследствие дефекта развития глоточных дуг или аутоиммунного поражения щитовидной железы в результате нарушения продукции Т-клеток и также является одним из характерных проявлений синдрома. По различным данным гипотиреоз встречается у 0,7–9,5% пациентов с синдромом делеции 22q11.2, гиперфункция щитовидной железы описана примерно в 1,8% случаев [3][9][27][28].

Выраженность клинических проявлений может варьировать от носительства антител без нарушения тиреоидного статуса до тяжелого гипо- или гипертиреоза, что мы наблюдали в нашем клиническом случае [29].

Помимо аутоиммунного поражения щитовидной железы, нарушения Т-клеточного звена иммунитета у пациентов с синдромом del 22q11.2 могут быть ассоциированы с такими аутоиммунными заболеваниями, как бронхиальная астма, воспалительные заболевания кишечника (ВЗК), ЮРА, аутоиммунные гемолитические анемии и т.д. [30].

Задержка роста у пациентов с синдромом del 22q11.2 описана в 36–41% случаев и чаще ассоциирована со ЗВУР, конституциональной задержкой роста (КЗР), декомпенсацией ВПС или нарушением питания в результате врожденных аномалий развития носоглотки [31][32][33]. Наличие дефицита гормона роста у пациентов с синдромом del 22q11.2 описано приблизительно в 4% случаев, что необходимо учитывать при проведении дифференциальной диагностики причин задержки роста и выбора оптимальной тактики ведения таких больных [3][34].

Исследование, проведенное с 1994 по 2020 гг. среди тайванских детей с синдромом del 22q11.2, продемонстрировало более выраженную задержку роста у пациентов первых 10 лет жизни по сравнению со старшей возрастной группой [35].

Так, оценка стандартного отклонения роста (SDS роста) у 27 пациентов в возрасте младше трех лет составила -2,14±2,56, у 41 пациента в возрасте от трех до десяти лет -2,05±1,53, у 24 пациентов в возрасте от десяти до восемнадцати лет -1,33±1,07 и -1,19±1,03 у 34 пациентов в возрасте старше 18 лет (P<0,05) [35]. Такая модель роста может быть частично объяснена ЗВУР (25 из 138 пациентов (18%)), КЗР или нарушением статуса питания из-за множественных врожденных аномалий, которые улучшались с возрастом [32][33]. Тем не менее конечный рост пациентов в исследуемой группе был ниже целевого, что соответствовало предыдущим сообщениям [24]. Дефицит гормона роста в данном исследовании не был выявлен [35].

Впервые наличие СТГ-дефицита у 4 пациентов с del 22q11.2 было описано в 1998 г. SA Weinzimer и соавт. [34]. Пациенты имели задержку роста >-2,0 SDS, низкий уровень ИФР-1 и отсутствие нутритивных нарушений. У одного из 4 пациентов, по данным МРТ головного мозга, была выявлена гипоплазия передней доли гипофиза, у другого — гипоплазия передней доли гипофиза в сочетании с эктопией нейрогипофиза. Катамнестическое наблюдение за пациентами в течение 2 лет выявило устойчивое улучшение скорости роста у трех детей, получавших терапию рекомбинантным гормоном роста (рГР). Родители 4-го пациента от терапии соматропином отказались [34].

Впоследствии СТГ — дефицит у пациентов с синдромом del 22q11.2 был описан еще рядом авторов [36][37].

Основные клинические проявления синдрома Ди Джорджи представлены в таблице 2.

Синдром Ван-Вика–Громбаха (ВВГ) представляет собой редкое эндокринное расстройство, при котором тяжелый первичный гипотиреоз приводит к симптомам преждевременного полового созревания. Впервые это состояние было зафиксировано еще в 1905 году, [38] однако ранние описания ограничивались клиническими наблюдениями. Лишь спустя более полувека, в 1960 году, исследователи J. Van Wyk и M. Grumbach предложили научное объяснение данного феномена. Согласно их гипотезе, ключевым механизмом развития синдрома является гормональный перекрест на уровне гипофиза: при выраженном гипотиреозе избыток тиролиберина (ТРГ) стимулирует не только синтез ТТГ, но и аномальную секрецию пролактина и гонадотропинов. Дополнительным фактором служит замедленный метаболизм эстрогенов, что усиливает их воздействие на ткани-мишени [39].

Со временем синдром ВВГ стал предметом многочисленных исследований, а его клинические и биохимические особенности были детально изучены в зарубежной и отечественной медицинской литературе [40][41][42].

В нашем клиническом случае поводом для обращения к эндокринологу послужило раннее появление вторичных половых признаков на фоне тяжелого первичного гипотиреоза в результате ХАИТ. У пациентки отсутствовали такие признаки синдрома делеции 22 хромосомы, как гипопаратиреоз и первичный иммунодефицит, что отсрочило своевременную постановку диагноза.

Учитывая анамнез заболевания, фенотипические особенности ребенка и полученные клинико-лабораторные данные, дифференциальный диагноз проводился между синдромами, сопровождающимися задержкой роста, лицевым дисморфизмом и ВПР, включая аномалии развития сердца и атрезию ануса. Среди возможных диагнозов рассматривались синдром Кабуки, синдром Вильямса, CHARGE-синдром, синдром Нунан и синдром Смита-Лемли-Опица (табл. 3). В связи с отсутствием у пациентки характерных фенотипических признаков, типичных для перечисленных синдромов, основным предположением стал синдром делеции 22-й хромосомы. Данный диагноз впоследствии был подтвержден результатами молекулярно-генетического исследования.

**Table:** Таблица 3. Дифференциальная диагностика синдрома делеции 22-й хромосомы [43][44][45][46][47]

Синдром	Генетическая причина заболевания	Фенотипические особенности	Эндокринные проявления	Прочие проявления
CHARGE синдром	Мутации в гене CHD7	Квадратное, асимметричное лицо, плоская средняя часть лица, выступающий лоб, изогнутые брови, большие глаза, птоз, выступающая переносица, маленькие ноздри, маленький рот и подбородок, расщелина губы и/или неба	Гипогонадотропный гипогонадизм, гипотиреоз, низкорослость, дефицит гормона роста, гипопаратиреоз	ВПС, колобома, сенсоневральная потеря слуха, атрезия хоан, задержка развития, трахеопищеводный свищ/атрезия пищевода, аномалии почек, сколиоз
Синдром Вильямса	Делеция длинного плеча хромосомы 7q11.23 (ELN)	Широкий лоб, периорбитальная полнота, звездчатый/кружевной рисунок радужки, косоглазие, короткий нос, широкий кончик носа, уплощение скул, толстая красная кайма губ, широкий рот, неправильный прикус, микрогнатия, большие мочки ушей	Низкорослость, ППР, гиперкальциемия, ожирение, гипотиреоз, нарушения углеводного обмена	ВПС, умственная отсталость, СДВГ, аномалии соединительной ткани, проблемы с ЖКТ, аномалии зубов
Синдром Нунан	Нарушения в системе сигнального пути RAS-MAPK (KRAS, SOS1, RAF1, NRAS, BRAF, SHOC2, CBL, RIT1)	Треугольная форма лица, короткая шея, крыловидные складки кожи на шее, низкий рост волос, гипертелоризм, птоз, низко посаженные и отогнутые назад уши, врожденная деформация локтевых суставов, готическое небо	Низкорослость, гипотиреоз, гипогонадизм, задержка полового развития, крипторхизм	ВПС, умственная отсталость, РАС, СДВГ, пороки развития мочеполового тракта, лимфедема, гематологические проявления, злокачественные новообразования
Синдром Кабуки	Мутация генов КМТ2D, KDM6A	Длинные глазные щели, выворот боковой трети нижнего века, изогнутые и широкие брови с редкой боковой третью, широкий/вдавленный кончик носа с короткой колумеллой, большие, выступающие или чашевидные уши	Низкорослость, ППР, ожирение в подростковом возрасте, гиперинсулинизм, надпочечниковая недостаточность, гипопитуитаризм, несахарный диабет, гипотиреоз, первичная дисфункция яичников	ВПС, умственная отсталость, потеря слуха, ПИД, аномалии почек и мочевыводящих путей, дефекты скелета, неврологическая, офтальмологическая симптоматика
Синдром Смита-Лемли-Опица	Биаллельные мутации в гене DHCR7	Микроцефалия, короткая шея с излишками кожи на затылке, битемпоральное сужение, птоз, укороченный нос с вывернутыми вперед ноздрями, микрогнатия, расщелина губы или неба, фокомелия, постаксиальная полидактилия, эктродактилия или синдактилия 2, 3 пальцев ног	Пренатальная и/или постнатальная задержка роста, первичная надпочечниковая недостаточность, гипоспадия, крипторхизм, нарушение формирование пола при кариотипе XY	ВПС, различные степени умственная отсталость, СДВГ, РАС, врожденная катаракта, атрофия зрительного нерва, аномалии мозга, почечные кисты, пилоростеноз, аганглионарный мегаколон, холестатическое заболевание печени

Генетическая верификация диагноза позволила составить дальнейший план ведения пациентки, чтобы своевременно скорректировать такие частые компоненты синдрома, как иммунная дисфункция и гипокальциемия, что потенциально снизит риск развития жизнеугрожающих осложнений.

## ЗАКЛЮЧЕНИЕ

Данный клинический случай демонстрирует вариабельность клинической симптоматики у пациентов с синдромом делеции 22-й хромосомы, когда отсутствие таких ведущих проявлений заболевания, как ПИД и гипопаратиреоз, не позволило заподозрить диагноз клинически на протяжении 8,5 года наблюдения до манифестации ППР на фоне декомпенсированного гипотиреоза. Привлечение мультидисциплинарной команды к наблюдению за пациентами с множественными ВПР и особенностями фенотипа, а также широкое внедрение методов генетического тестирования в повседневную клиническую практику позволит заподозрить заболевание до развития тяжелых осложнений синдрома и разработать персонализированный план ведения таких больных.

## ДОПОЛНИТЕЛЬНАЯ ИНФОРМАЦИЯ

Источники финансирования. Работа выполнена по инициативе авторов без привлечения финансирования.

Конфликт интересов. Авторы декларируют отсутствие явных и потенциальных конфликтов интересов, связанных с содержанием настоящей статьи.

Участие авторов. Все авторы одобрили финальную версию статьи перед публикацией, выразили согласие нести ответственность за все аспекты работы, подразумевающую надлежащее изучение и решение вопросов, связанных с точностью или добросовестностью любой части работы.

Согласие пациента. Законный представитель пациента добровольно подписал информированное согласие на публикацию персональной медицинской информации в обезличенной форме в журнале «Проблемы эндокринологии».
